# PReS13-SPK-1575: Science of pain amplification

**DOI:** 10.1186/1546-0096-11-S2-I35

**Published:** 2013-12-05

**Authors:** RF Howard

**Affiliations:** 1 Anaesthesia and Pain Medicine, Great Ormond St Hospital For Children, London, UK; 2 UCL Paediatric Pain Research Group, University College London, London, UK

## 

Pain perception is subject to powerful modulatory influences that can increase or reduce subjective responses in the short and long term. Nociceptive circuits can become sensitised causing both innocuous and noxious inputs to be amplified and their intensity and other sensory characteristics to be increased. Both central and peripheral mechanisms of sensitisation are recognised. Nociceptive mechanisms are immature at birth, undergoing both structural and functional change during normal development that can have important consequences for the maintenance, management and prognosis of chronic pain in children.

The neurophysiology of pain in children will be briefly described with special reference to sensitisation in the context of non-inflammatory musculoskeletal pain and pain amplification syndromes. A mechanisms based approach to the medical management of chronic pain in children will be discussed.

## Disclosure of interest

None declared.

